# Covered TIPS Procedure-Related Major Complications: Incidence, Management and Outcome From a Single Center

**DOI:** 10.3389/fmed.2022.834106

**Published:** 2022-05-04

**Authors:** Xiaochun Yin, Lihong Gu, Ming Zhang, Qin Yin, Jiangqiang Xiao, Yi Wang, Xiaoping Zou, Feng Zhang, Yuzheng Zhuge

**Affiliations:** Department of Gastroenterology, Nanjing Drum Tower Hospital, The Affiliated Hospital of Nanjing University Medical School, Nanjing, China

**Keywords:** transjugular intrahepatic portosystemic shunt, portal hypertension, major complications, covered stents, hemobilia

## Abstract

**Background and Objective:**

Transjugular intrahepatic portosystemic shunt (TIPS) is a well-established procedure for treating complications of portal hypertension. Due to the complexity of anatomy and difficulty of the puncture technique, the procedure itself might brought potential complications, such as puncture failure, bleeding, infection, and, rarely, death. The aim of this study is to explore the incidence, management, and outcome of TIPS procedure-related major complications using covered stents.

**Methods:**

Patients who underwent TIPS implantation from January 2015 to December 2020 were recruited retrospectively. Major complications after TIPS were screened and analyzed.

**Results:**

Nine hundred and forty-eight patients underwent the TIPS procedure with 95.1% (n = 902) technical success in our department. TIPS procedure-related major complications occurred in 30 (3.2%) patients, including hemobilia (*n* = 13; 1.37%), hemoperitoneum (*n* = 7; 0.74%), accelerated liver failure (*n* = 6; 0.63%), and rapidly progressive organ failure (*n* = 4; 0.42%). Among them, 8 patients died because of hemobilia (*n* = 1), accelerated liver failure (*n* = 4), and rapidly progressive organ failure (*n* = 3).

**Conclusion:**

The incidence of major complications related to TIPS procedure is relatively low, and some of them could recover through effective medical intervention. In our cohort, the overall incidence is about 3%, which causes 0.84% death. The most fatal complication is organ failure and hemobilia.

## Introduction

Transjugular intrahepatic portosystemic shunt (TIPS) is a well-established procedure for treating complications of portal hypertension, including variceal bleeding, refractory ascites, hepatic sinusoidal obstruction syndrome (HSOS), hepatorenal syndrome, portal vein thrombosis, and Budd-Chiari syndrome (1). Previous studies have reported that stent dysfunction, re-bleeding, and hepatic encephalopathy (HE) were common complications following TIPS placement, which have restricted the clinical application of TIPS (2, 3). However, the efficacy has been improved, benefiting from the use of covered stents. In recent years, the indication for TIPS has been changed and TIPS surgery is more widely carried out.

The TIPS procedure is performed by placing a stent to create a large channel between the portal vein and the hepatic vein in the liver parenchyma, allowing for brisk blood flow through the liver and reducing portal pressure. Due to the complexity of anatomy and e difficulty of the puncture technique, the procedure itself might bring potential complications, such as puncture failure, bleeding, infection, and, rarely, death. Over the past 30 years, a number of complications have been identified. It is reported that direct procedure-related complication rates are as high as 20% (4). However, most were minor complications, which were not serious and did not affect survival.

Major complications during the TIPS procedure are often fatal, such as bleeding, infection, continuous heart or kidney failure, accelerated liver failure, and vascular and bile duct damages (5), which result in an unplanned increase in the level of care, prolonged hospitalization, or death (6). Thus, clinicians require a thorough stepwise understanding of TIPS insertion, strictly grasp the indications to select patients, and follow up closely after TIPS to minimize the major complications. The incidence needs to be revealed. Although the reported risk for major complications is up to 3% and the procedural mortality is 1.7% (7), these data are often based on comprehensive review of bare stents (8), and cohort studies are rare. Since TIPS has already entered the era of covered stents, it is urgent to rediscover major complications in a large cohort.

This study was designed to explore the incidence, management, and outcome of TIPS procedure-related major complications using covered stents in a cohort of patients with portal hypertension, focusing on early fatal complications.

## Methods

### Patients

The patients in this study were screened from the prospective database of the Department of Gastroenterology in Nanjing Drum Tower Hospital. A cohort of 948 TIPS procedures for patients with portal hypertension from January 2015 to December 2020 was reviewed. Perioperative data and patients' clinical outcomes were obtained to determine whether these patients suffered major complications, which were defined according to Quality Improvement Guidelines for TIPS (6). According to the guidelines, we analyzed data from patients suffered hemoperitoneum, biliary injury, stent malposition, radiation skin burn, hepatic infarction, rapidly progressive organ failure (including lung, heart and kidney), hepatic artery injury, accelerated liver failure, severe or uncontrolled encephalopathy, death, and other rare but fatal complications. Written informed consent was obtained from each patient before TIPS. The study protocol conformed to the principles of the 1975 Declaration of Helsinki.

### Preoperative Examination

Complete blood routine, liver function, kidney function, coagulation function, electrolytes, and other laboratory tests were performed before TIPS. Abdominal CT portal vein reconstruction helped understand the anatomy of the patient's liver and blood vessels. For those who were suspected of having heart disease, ECG and heart color Doppler ultrasound were performed to rule out severe heart failure. Puncture and drainage were performed before TIPS for those with a large amount of ascites or pleural fluid.

### TIPS Procedure

All the procedures were completed by 2–3 experienced gastroenterology intervention doctors in this institution. The process of the TIPS procedure has been described in detail in previous articles (9). Briefly, the right or middle hepatic vein was catheterized using a transjugular venous approach with RUPS-100 (COOK; Indiana, United States) under local anesthesia. Indirect portal venography was performed through the superior mesenteric artery or the splenic artery. After showing the portal vein, the portal vein from the hepatic vein was punctured. After successful puncture, a 6–8-mm diameter covered stent (Fluency; Bard, New Jersey, United States or Viatorr; W.L. Gore & Associates, Arizona, United States) or a covered stent combined with a bare metal stent (Fluency; Bard with Luminexx; Bard), was deployed into the tract to support the parenchymal channel.,Then the stent was dilated with a balloon catheter. If evident stomach and esophageal varices presented, embolization was performed to fill the residual varices with coils or tissue-adhesive glue. Portal pressure gradient (PPG) was measured before and after the shunt procedure. All the operations were completed by the same intervention team. Blood pressure and heart rate were measured during operation.

### Postoperative Treatment

Continue to monitor vital signs after TIPS for at least 24 h, including blood pressure, heart rate, peripheral circulation status and 24-h urine output. Observe whether there is blood oozing and hematoma formation at the arterial and venous puncture points. Check Hb/hematocrit, liver function, kidney function and electrolytes 24 h after TIPS. An appropriate amount of hepatoprotective drugs were used. Reduce the amounts of diuretics based on urine output and clinical symptoms. According to the thrombosis of the portal vein system during the operation, the catheter was indwelled, and thrombolytic therapy with low molecular-weight heparin and urokinase was given. Complete Doppler ultrasound examination before discharge to observe blood flow in the portal vein and the stent to rule out stenosis.

### Clinical Outcome Definitions

TIPS-hemoperitoneum often occurs during puncture of the intrahepatic portal vein branch, puncturing the liver capsule and mispenetrating or injuring the extrahepatic portal vein to form a peritoneal shunt. The patient suddenly presents with a rapid heart rate, a sudden drop in blood pressure, and even hemorrhagic shock. It can be diagnosed by portal angiography and paracentesis.

TIPS-hemobilia is defined as intrahepatic bile duct bleeding due to puncture injury or shunt obstruction of the intrahepatic bile duct. Clinically significant hemobilia presents with biliary colic, jaundice, and gastrointestinal bleeding after TIPS placement, and may range from occult to massive bleeding. The diagnosis of hemobilia is confirmed by upper endoscopy or computed tomography angiography (CT-A) (10).

Rapidly progressive organ failure mainly presented with lung, heart, or kidney insufficiency, which was progressively aggravated after TIPS. Increased cardiac preload and pulmonary artery pressure following TIPS could contribute to the consequences of this phenomenon.

Accelerated liver failure was defined as ≥3-fold bilirubin and/or ≥2-fold INR elevation associated with clinical outcomes of prolonged hospitalization/increase in care level, liver transplantation, or death within 30-days of TIPS (11).

### Statistical Analysis

The data of this study were processed with the SPSS 22.0 software, the incidence and mortality of different major complications within 1 month after TIPS were analyzed, and the count data were expressed in %.

## Results

### Patients' Baseline Characteristics

**Figure 1:**
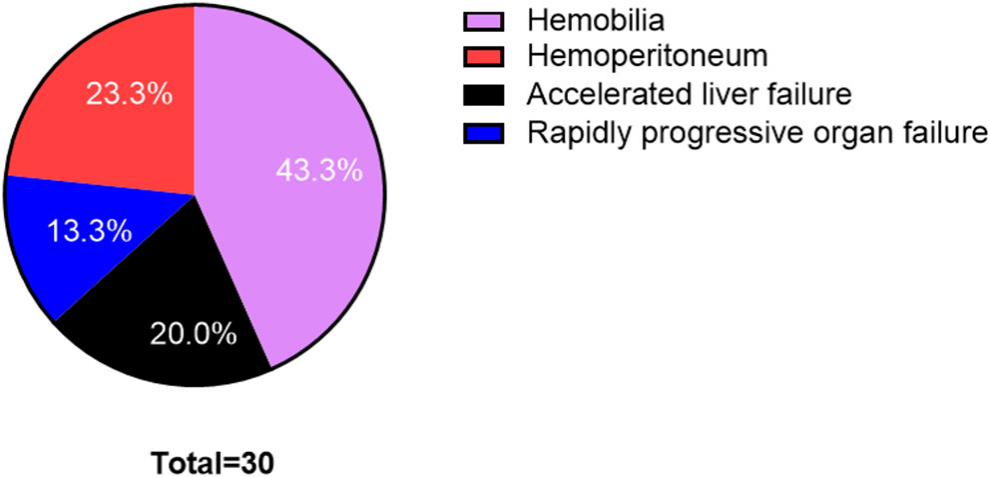
Proportion figure of postoperative complications in transjugular intrahepatic portosystemic shunt (TIPS) procedure.

From 2015 to 2020, 948 patients underwent the TIPS procedure with 95.1% (*n* = 902) technical success in our department. TIPS procedure-related major complications occurred in 30 (3.2%) patients. Technical-related major complication rates every year were 2.6 (3/113), 2.7 (4/148), 4.3 (6/138), 5.4 (9/164), 2.4 (5/207), and 1.7% (3/174) from 2015 to 2020, respectively. Patients' baseline characteristics are shown in Table 1. Seventeen patients received TIPS for liver cirrhosis, and 9 for HSOS, and 4 for Budd-Chiari syndrome. The major complications included hemobilia, hemoperitoneum, accelerated liver failure, and rapidly progressive organ failure (Table 2, Figure 1). Eight patients (0.84%) died because of major complications.

**Table 1:** Patients' clinical characteristics.

**Variables**	**Patient population (*N* = 30)**
Age (yr)	63 (15–78)
Gender: male [*n* (%)]	14 (46.7%)
Etiology: Cirrhosis/ HSOS/ Others	17/9/4
Indication for TIPS:bleeding/ascites/PVT	18/9/3
CTP.score	8 (5–13)
CTP class (A/B/C)	4/17/9
MELD score	11 (6–35.6)
PT (s)	14.9 (11.4–46.5)
INR	1.3 (1.0–4.1)
TBil (umol/L)	32.9 (4.8–153.6)
Albumin (g/L)	31.9 (19.5–37.6)
Scr (ummol/L)	67.5 (33.0–350.0)
WBC (×10^∧^9/L)	4.9 (0.7–13.2)
PLT (×10^∧^9/L)	74.5 (4.0–359.0)
Ascites (No/Light/Medium/Heavy)	3/5/13/9
Stent diameter (6/7/8 mm)	4/3/23

*Data are expressed as mean, number (%), or median (range)*.

*HSOS, hepatic sinusoidal obstruction syndrome; CTP, Child-Turcotte-Pugh; MELD, model of end-stage liver disease; PT, prothrombin time; INR, international normalized ratio; TBil, total bilirubin; Scr, serum creatinine; WBC, white blood cells; PLT, platelet*.

**Table 2:** Major complications of transjugular intrahepatic portosystemic shunt (TIPS) [*n* (%)].

**Complication**	**Cases (%)**	**Death**	**Reported rate (%) (6)**	**Suggested threshold (%) (6)**
Hemobilia	13 (1.37)	1 (7.6)	2	2
Hemoperitoneum	7 (0.74)	0 (0)	0.5	1
Accelerated liver failure	6 (0.63)	4 (66.7)	3	-
rapidly progressive organ failure	4 (0.42)	3 (75.0)	0.25^*^	0.5
Biliary peritonitis	0	0	1	2
Stent malposition	0	0	1	1
Radiation skin burn	0	0	0.1	0.1
Hepatic infarction	0	0	0.5	0.5
Hepatic artery injury	0	0	1	2
Total	30 (3.2)	8 (0.84)	3	5

* *0.25% is the rate of renal failure requiring chronic dialysis*.

### Hemobilia

**Figure 2:**
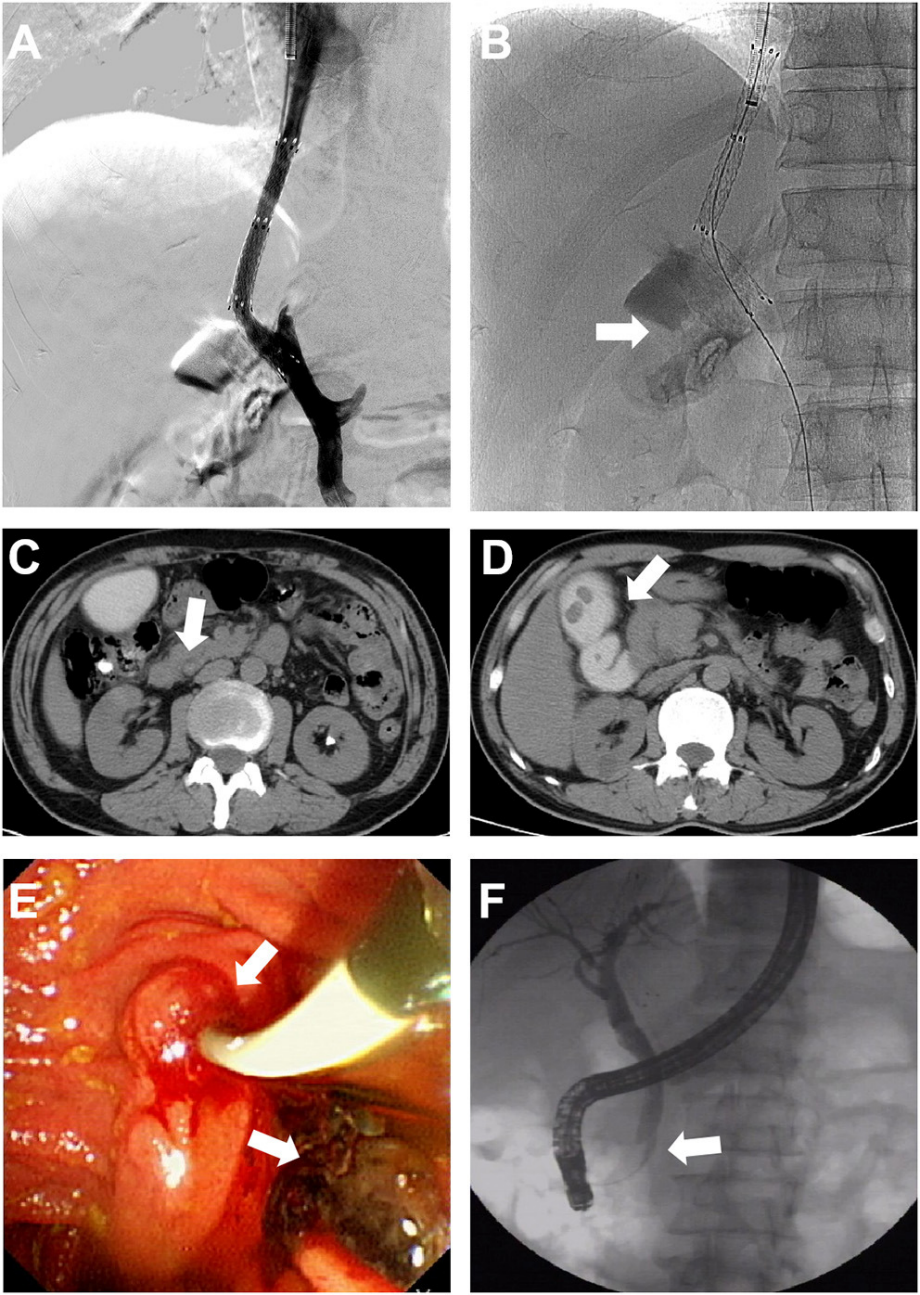
Characteristics and management of hemobilia. **(A,B)** Bile duct was punctured during the procedure and contrast medium retention was found in the gallbladder. **(C,D)** Serum bilirubin elevated gradually after TIPS. CT showed bile duct dilatation, retention of contrast medium in the gallbladder, and high-density shadow of the common bile duct. **(E,F)** Endoscopic retrograde cholangiography (ERCP) showing a filling defect in the common bile duct, and the bleeding clot was cleared. The nasobiliary duct was retained for drainage.

Thirteen (1.37%) patients developed hematemesis, abdominal pain, and obstructive jaundice after TIPS owing to biliary injury during the procedure, including 10 patients with liver cirrhosis and 3 cases with HSOS. Only 3 patients recovered with conservative medicine treatment. Eight patients underwent endoscopic retrograde cholangiography (ERCP), bile duct cleaning, and nasobiliary drainage (Figure 2); one patient underwent percutaneous transhepatic biliary drainage, and 1 patient underwent hepatic artery embolization. Finally, one patient died because of continuous bleeding.

### Hemoperitoneum

Seven patients (0.74%) suffered shock caused by hemoperitoneum within 24 h after the TIPS procedure (Table 3). Five patients had portal vein thrombosis (PVT), which often needed anticoagulant and thrombolytic therapy. Two patients received emergency surgical operation to repair the injured main portal vein and achieved successful hemostasis. Five patients recovered from intraperitoneal puncture and continuous drainage and medical treatment. No patients died.

**Table 3:** Patients who had hemoperitoneum.

**Cases**	**Etiology**	**PVT**	**Anticoagulant and thrombolytic therapy**	**Speicial treatment**
Case 1	Cirrhosis	No	No	Drainage
Case 2	Cirrhosis	Yes	Yes	Surgery
Case 3	Cirrhosis	Yes	No	Drainage
Case 4	Cirrhosis	Yes	Yes	Drainage
Case 5	Cirrhosis	Yes	No	Drainage
Case 6	Non-cirrhotic PVT	Yes	No	Surgery
Case 7	HSOS	No	Yes	Drainage

*Anticoagulant and thrombolytic therapy included heparin and urokinase. PVT, Portal vein thrombosis*.

### Accelerated Liver Failure

Accelerated liver failure was defined as ≥3-fold bilirubin and/or ≥2-fold INR elevation associated with clinical outcomes of prolonged hospitalization/increase in care level, liver transplantation, or death within 30-days of TIPS (11). Rapidly accelerated liver failure occurred in 6 patients (0.63%), accompanied with uncontrolled encephalopathy, who were given artificial liver support therapy. However, 4 patients died.

### Rapidly Progressive Organ Failure

Rapidly progressive organ failure occurred in 4 patients. Three of them died because of uncontrolled respiratory and heart failure. Two of them had idiopathic portal hypertension caused by systemic lupus erythematosus (SLE) and acute portal hypertension caused by HSOS, and both had refractory ascites and severe renal insufficiency before the TIPS procedure. Of the 4 patients, only one patient recovered spontaneous vital signs after active rescure.

## Discussion

In this study, we reported the incidence, management, and outcome of covered TIPS procedure-related major complications in a large cohort of patients from one single center for the first time. The overall incidence of major complications in our cohort was 3.2%, which was consistent with the reported rate and lower than the suggested threshold (6). A previous study has shown that complications including liver and biliary system damage and abdominal hemorrhage occur 10–15% of the time during the era of bare stents (12).

The application of TIPS in the treatment of portal hypertension has been widely accepted; however, complex and diverse complications during or after the procedure remain a problem. TIPS is still one of the most complicated and technically difficult interventional procedures; especially, endovascular punctures of the portal vein are the main reason for perioperative complications and TIPS failure. In recent years, the technical success of TIPS creation has been reported to be over 95%, which is consistent with this study. In our view, patency of the portal vein is the key to TIPS technical success. In our cohort, the success rate was close to 100% in patients without portal vein embolism but lower than 90% in patients with portal vein cavernous transformation.

Hemobilia is the most common major complication in this study, and is observed in 13 (1.37%) patients. During TIPS procedure, puncture of the biliary tract cannot be completely avoided, and the incidence rate exceeds to 1.37%. However, most of its cases do not need additional attention and treatment. A previous study involving 135 cirrhotic patients who underwent TIPS identified 4 cases (2.9%) of segmental intrahepatic cholestasis (13). The clinical manifestation of hemobilia might be continuous bleeding and even shock or obstructive jaundice caused by blood clot (14, 15). Both could be well-controlled with endoscopic or percutaneous drainage, embolization, and medical therapy. Often, hemobilia is a self-limiting phenomenon, and expectant observation is a commonly used option. Therefore, only 1 patient (7.6%) died because of hemobilia. In addition, angiography with embolization is the treatment of choice for most cases of hemobilia to stop the bleeding and restore bile flow.

The most fatal complication is accelerated liver failure. Although it only occurred in 6 (0.63%) patients, 4 (66.7%) died. These patients were accompanied with uncontrolled encephalopathy. In previous studies, early post-TIPS liver failure is a common complication (2, 16). TIPS results in acute transient derangement of liver function, which may be more pronounced in patients with early mortality (17, 18). Regardless of TIPS indication, patients with very advanced liver disease (Child-Pugh >13) tend to have poor survival. Liver failure could also be due to segmental hepatic ischemia induced by a covered TIPS stent (19). The reported incidence was up to 3%, which was much higher than in our cohort. However, this is highly dependent on patient selection. Most of the patients in this cohort underwent TIPS for control of variceal bleeding and usually had better liver function. When patients develop accelerated liver failure after TIPS, they should be treated with plasma exchange therapy as soon as possible to restore liver function. In our study, 3 patients were treated with plasma exchange. When patients develop refractory hepatic encephalopathy or accelerates liver failure after TIPS, shunt restriction or closure should be performed. In addition, alternative treatments including endoscopic sclerotherapy, variceal ligation and large-volume paracentesis can be used. Finally, liver transplantation is the treatment of choice (20).

Over the past years, hemoperitoneum has been a fatal complication during TIPS, with an incidence of about 0.6–4.2% (4, 7). Most of the procedure-related mortality is related to hemorrhagic complications, which varies from <1–1.2%. When the extrahepatic portal vein is punctured, bleeding might be much massive. However, because of the use of covered stents, this has been significantly decreased. Another reason for hemoperitoneum is liver capsule puncture and damage. Extracapsular penetration can lead to rapid development of hemoperitoneum, particularly in patients receiving anticoagulant and thrombolytic therapy, which might explain the higher rate of hemoperitoneum (0.74%) in our cohort. In our study involving TIPS with covered stents, all cases of hemoperitoneum caused by liver capsule damage were cured with intraperitoneal puncture and continuous drainage, which was simple, safe, efficient and economical.

Rapidly progressive organ failure involving the lung, heart, and kidneys seemed to be also fatal in our cohort, and caused 3 deaths in 4 patients with refractory ascites. There have also been reports of significant cardiorespiratory complications after TIPS (21). Increased cardiac preload and pulmonary artery pressure following TIPS could contribute to the consequences of this phenomenon. Therefore, patients with water and sodium retention should be checked for congestive heart failure and pulmonary hypertension before the procedure to avoid exacerbation of cardiac insufficiency after TIPS.

In total, 8 patients died because of major complications, which is comparable with previous studies. However, the composition might be different. In this cohort, 7 patients died because of organ failure including liver, lung, and heart, and only one patient died because of hemobilia. TIPS creation is still one of the most challenging procedures performed by interventional radiologists. It reported that the technical success rate of TIPS should be greater than 95%, and the major complication rate is less than 5% with increased experience (22). In our center, only a few major complications were included. However, according to Quality Improvement Guidelines for TIPS (6), stent malposition, radiation skin burn, liver infarction and hepatic artery injury are also major complications after TIPS. Besides, other complications such as cardiac perforation due to stent embolization (23), endotipsitis-persistent infection of TIPS stent (24), and mechanical hemolysis (25) were also not observed.

## Conclusion

With the continuous development of TIPS and technological innovation, the incidence of major complications related to the procedure is relatively low, but causes are diverse and risk factors are mixed. In our center, the overall incidence is about 3%, which could cause 0.84% death. The most fatal complications are organ failure and hemobilia.

## Data Availability Statement

The raw data supporting the conclusions of this article will be made available by the authors, without undue reservation.

## Ethics Statement

The studies involving human participants were reviewed and approved by the Ethics Committee of the Nanjing Drum Tower Hospital, Nanjing, Jiangsu, China. The patients/participants provided their written informed consent to participate in this study. Written informed consent was obtained from the individual(s) for the publication of any potentially identifiable images or data included in this article.

## Author Contributions

XY, LG, and FZ contributed to the conception and design of the study. XY and QY organized the database. XY and MZ performed the statistical analysis. XY, XZ, and FZ wrote the first draft of the manuscript. MZ, YW, QY, and JX wrote sections of the manuscript. All the authors contributed to manuscript revision, read, and approved the submitted version.

## Funding

This study was supported by the National Natural Science Foundation of China (No. 8190032307) and Nanjing Health Science and Technology Development Special Fund Project-Outstanding Youth Fund project (JQX20005).

## Conflict of Interest

The authors declare that the research was conducted in the absence of any commercial or financial relationships that could be construed as a potential conflict of interest.

## Publisher's Note

All claims expressed in this article are solely those of the authors and do not necessarily represent those of their affiliated organizations, or those of the publisher, the editors and the reviewers. Any product that may be evaluated in this article, or claim that may be made by its manufacturer, is not guaranteed or endorsed by the publisher.
